# Sir Felix Schuster on Hospital Finance

**Published:** 1913-11-29

**Authors:** 


### Great Northern Hospital.

SIR FELIX SCHUSTER ON HOSPITAL FINANCE.

Sir Felix Schuster presided on Thursday, last week, at a dinner at the Whitehall Rooms in aid of the Great Northern Central Hospital. In proposing success to the institution he said they knew the number of appeals which every post brought, and they knew they could not satisfy them all. A very eminent physician had remarked to him before he came there that day that the hospital was the best in London. It was the only general hospital in the largest borough of London—Islington with its 320,000 inhabitants—66 per cent. of its in- and out-patients came from that borough. But it appealed to a much wider area than Islington. In 1912 no fewer than 178 in-patients from districts entirely outside the metropolitan area were admitted, at a cost to the hospital of £1,265, and that alone afforded justification for the hospital to extend its appeals to the country no less than to London. In 1912 they had 185 beds available, and in those beds they treated 2,517 persons. The out-patients numbered 23,484. The income amounted to £16,900, which was £3,200 less than the expenditure. It was serious that the great work performed by hospitals of that sort should be so much at the mercy of circumstances and depend almost for its existence upon appeals of that kind. But many were of opinion that the voluntary system was better for the public, the nation, and the medical profession, than the State-supported hospitals of foreign countries. He thought the general feeling was against State-supported hospitals.

He had not a word to say about the fund collected in connection with the Crystal Palace, but when it was possible to collect such a sum for recreation he thought a smaller sum with which they could be contented ought not to be too difficult to obtain, not for recreation, but for the restoration of health. He believed in individuals trying to provide for themselves rather than trusting to the State. Hospitals endeavoured as far as they could to assist their brothers and sisters back to health in order that they might make good and useful citizens.

The toast was responded to by Mr. H. J. Tennant, M.P., and Dr. Alexander Morison. Donations were announced amounting altogether to £7,488.

